# A Quantitative and Novel Approach to the Prioritization of Zoonotic Diseases in North America: A Public Perspective

**DOI:** 10.1371/journal.pone.0048519

**Published:** 2012-11-01

**Authors:** Victoria Ng, Jan M. Sargeant

**Affiliations:** Centre for Public Health and Zoonoses, Department of Population Medicine, Ontario Veterinary College, University of Guelph, Guelph, Canada; The Scripps Research Institute Scripps Florida, United States of America

## Abstract

**Background:**

Zoonoses account for over half of all communicable diseases causing illness in humans. As there are limited resources available for the control and prevention of zoonotic diseases, a framework for their prioritization is necessary to ensure resources are directed into those of highest importance. Although zoonotic outbreaks are a significant burden of disease in North America, the systematic prioritization of zoonoses in this region has not been previously evaluated.

**Methodology/Principal Findings:**

This study describes the novel use of a well-established quantitative method, conjoint analysis (CA), to identify the relative importance of 21 key characteristics of zoonotic diseases that can be used for their prioritization in Canada and the US. Relative importance weights from the CA were used to develop a point-scoring system to derive a recommended list of zoonoses for prioritization in Canada and the US. Over 1,500 participants from the general public were recruited to complete the online survey (761 from Canada and 778 from the US). Hierarchical Bayes models were fitted to the survey data to derive CA-weighted scores. Scores were applied to 62 zoonotic diseases of public health importance in Canada and the US to rank diseases in order of priority.

**Conclusions/Significance:**

This was the first study to describe a systematic and quantitative approach to the prioritization of zoonoses in North America involving public participants. We found individuals with no prior knowledge or experience in prioritizing zoonoses were capable of producing meaningful results using CA as a novel quantitative approach to prioritization. More similarities than differences were observed between countries suggesting general agreement in disease prioritization between Canadians and Americans. We demonstrate CA as a potential tool for the prioritization of zoonoses; other prioritization exercises may also consider this approach.

## Introduction

Zoonotic diseases are defined as those that are naturally transmitted between vertebrate animals and humans [1]. Zoonoses account for over half of all communicable diseases causing illness in humans [2], [3]. As there are limited resources available for research, surveillance, control and prevention of zoonoses, it is necessary to prioritize diseases in order to direct resources into those with the greatest needs. While there is general consensus amongst medical and veterinary professionals for the need to prioritize zoonoses, there are numerous challenges. Firstly, zoonotic diseases vary greatly in their occurrence and in their health impact on the human and animal populations, making it difficult to compare their overall public health importance [2]. Second, there is no universal agreement on the measurable criteria by which to quantify and prioritize zoonoses, nor is there agreement on the methodologies to elicit such information [4]–[6]. Lastly, there are a number of stakeholders involved, each with their own objectives [7]. It is therefore difficult to establish a universally accepted priority list for zoonotic diseases.

A number of studies have attempted to methodically prioritize communicable diseases of public health concern [6], [8]–[13]. With respect to zoonoses, a framework for their prioritization has been developed in France (non-foodborne zoonoses) [14], [15], Belgium (foodborne zoonoses) [16], The Netherlands (emerging zoonoses) [17] and Europe (food-producing animal diseases and zoonoses) [18]. Krause and colleagues [4] compared prioritization methodologies published between 1995 and 2005 and noted there was no uniformity in the objectives, methodological approaches, criteria for prioritization, number of criteria considered, level of standardization and weighting of criteria. Despite methodological differences, it is agreed that risk-based priority should be systematic, empirical and quantitative, easy to implement, based on good science, transparent, flexible, reproducible and informative to public policy [4], [19]. In recent studies, there has been a shift towards the use of novel quantitative approaches to overcome limitations in traditional methods and to address the complexity of disease prioritization [4], [6], [13], [16], [17].

Conjoint analysis (CA) has been used in market research of consumer preferences over the last 40 years [20]. More recently, this method has gained widespread use in the health and medical setting in eliciting preferences for healthcare [21]–[23]. The theory behind CA is that any product (goods or services) can be described by a set of characteristics and the extent to which an individual places value on a product is determined by the level of those separate characteristics (part-worth of the product) and the combination of those characteristics (overall worth of the product) [21], [22], [24]. CA forces individuals to make decisions by presenting competing products with both desirable and undesirable characteristics and asking individuals to state a preference. In expressing preference, individuals make a trade-off between the desirable and undesirable characteristics in those products allowing researchers to determine the true value of each characteristic relative to all other characteristics. A relative weighted score for each characteristic is derived as well as an overall score for each competing product as a whole. In the context of zoonoses, each disease can be treated as a product described by a set of disease criteria (characteristics), and the value of the disease is determined by the level of each criterion and the combination of the value of those levels. The overall score for each disease can be used to rank diseases in order of priority. Although there are similar methods to CA including Maximum Difference Scaling (MaxDiff) that has an emphasis on prioritizing a list of characteristics together [24], the complexity of zoonoses requires an understanding of preferences in the face of multiple levels of multiple characteristics combined together. Conjoint Analysis allows for the exploration of inter-relationships between various levels of disease characteristics together and was considered a more appropriate method for exploring preferences for disease characteristics and levels.

Current prioritization methods are limited by numerous challenges [4]–[6], [8]–[18], many of these can be overcome using CA, these include: (1) generating relative weighted scores; a better representation of criteria importance compared to arbitrary scores and subjective weights, (2) eliciting preferences through choice; an intuitive approach as opposed to ranking or rating diseases, (3) considering disease criteria jointly rather than separately; a more realistic approach to decision-making that recognizes disease criteria are not equal nor independent from each other, (4) presenting zoonoses as a set of disease characteristics without identifying diseases; this forces individuals to prioritize based on science thereby reducing biases associated with disease names, these can include the potential fear of a disease name compared to a lesser-known disease or prioritizing diseases based on professional interest and/or personal gain), and (5) presenting respondents with all the information necessary to prioritize diseases; this allows for prioritization to be based on wide social participation including public participation and experts who may not be familiar with the full range of diseases. We note in particular point number 4, by choosing to conduct an unlabeled prioritization study.

Although zoonotic outbreaks are a significant burden of disease in North America [25]–[27], the systematic prioritization of zoonoses in this region has not been previously evaluated. The primary objective of this study was to describe the novel use of a quantitative method, CA, to develop a point scoring system for disease criteria considered important in determining zoonotic disease priority. The second objective was to use the CA-derived scores to develop and compare a priority list of zoonoses in Canada and the US. This paper will focus on the results of individuals from the general public; this is the first study to describe a systematic and quantitative approach to the prioritization of zoonotic diseases from a public perspective.

## Methods

### Focus Groups and Criteria Identification

The authors previously described the six focus groups using the nominal group technique (NGT) to inform this study [7]. Individuals from the public and medical and veterinary professionals identified 59 unique disease criteria to prioritize zoonoses. Of these, 21 were used to inform the CA experimental design (Tables 1, 2, 3). These criteria were selected on the basis of having a high mean score (derived from the NGT) across groups, identified by three of more groups, or corresponding to human-related criteria that were deemed important (e.g. incidence in humans scored high, therefore, incidence in animals was included despite a lower score). A number of criteria were merged together, for example, *pathogenicity*, *immunogenicity*, *incubation period*, *communicability* and *mode of transmission* were integrated into *transmission potential*. This process allowed for a more efficient number of the most relatively important disease characteristics to be included in the study.

**Table 1:** Disease criteria and standardized part-worth utility values (β) for disease criteria levels by country.

Disease criteria^1^ and corresponding levels	Canada		US		*t* 4
	*MUV* 2	*SD* 3	*MUV* 2	*SD* 3	
*Case-fatality in humans*					
No deaths or deaths are rarely reported	−68.97	30.90	−89.60	29.97	13.30***
Case-fatality is low (6%)	−53.69	21.49	−45.59	20.23	7.62***
Case-fatality is moderate (35%)	20.59	22.78	22.02	26.43	1.14∧
Case-fatality is high (80%)	102.07	32.25	113.17	28.60	7.14*** ∧
*Incidence of the disease in the Canadian/US human population in the last five years*					
0 cases	−72.23	27.16	−85.10	31.02	8.66*** ∧
5 cases	−39.50	23.14	−37.25	22.11	1.95
100 cases	17.79	18.97	23.00	16.45	5.76*** ∧
10,000 cases	93.94	37.71	99.34	33.69	2.96** ∧
*Case-fatality in animals*					
No deaths or deaths are rarely reported	−61.85	27.48	−49.87	19.72	9.81*** ∧
Case-fatality is low (6%)	−27.10	17.61	−33.58	15.82	7.59*** ∧
Case-fatality is moderate (35%)	10.72	23.28	8.52	19.12	2.02* ∧
Case-fatality is high (80%)	78.23	29.56	74.93	22.65	2.46* ∧
*Incidence of the disease in the Canadian/US animal population in the last five years*					
0 cases	−57.44	30.36	−63.48	21.73	4.48*** ∧
5 cases	−27.41	21.62	−33.30	22.75	5.20***
100 cases	11.75	21.22	17.59	20.46	5.50***
10,000 cases	73.11	34.79	79.18	24.75	3.94*** ∧
*Severity of illness in humans*					
No clinical symptoms or illness that is not noticeable	−54.46	22.61	−54.64	26.54	0.15∧
Mild clinical symptoms (time off work, some medical assistance and personal care at home)	−28.07	23.11	−26.93	14.68	1.16∧
Moderate clinical symptoms (urgent medical care and hospital admission)	8.29	19.19	8.28	18.40	0.02
Severe clinical symptoms (failure of major organ system/s necessitating long-term hospital admission)	74.24	29.72	73.29	29.71	0.63
*Disease trend in Canada/US in the last five years in humans*					
Decline over the last five years	−58.29	24.03	−58.83	18.49	0.50∧
Stable over the last five years	−34.30	20.40	−26.89	18.53	7.45*** ∧
Increase over the last five years	29.29	19.26	26.43	17.85	3.02** ∧
New emerging disease, rapid increase over the last five years	63.30	26.80	59.29	24.37	3.07** ∧
*Transmission potential between humans*					
No transmission between humans	−52.93	22.50	−47.07	18.19	5.61*** ∧
Low transmission between humans	−30.86	19.95	−34.42	21.40	3.37**
Moderate transmission between humans	12.71	17.35	17.57	18.87	5.28*** ∧
High transmission between humans	71.08	18.90	63.92	21.28	6.98*** ∧

1 Presented in order of importance to Canadian participants.

2 Mean part-worth utility values (β) across respondents.

3 Standard deviation of mean part-worth utility values (β) across respondents.

4 *t*-statistic; *d.f*. = 1,537.

* *p*<0.05,

** *p*<0.01,

*** *p*<0.001.

∧ Adjusted for unequal variance using the Welch *t*-test; Satterthwaite’s *d.f.* = 1,282.61 to 1531.22.

Criteria that were difficult to quantify in measureable units (e.g. *ability of the disease pathogen to mutate*) or lacked scientific information for quantification (e.g. *bioterrorism potential*) were excluded. Criteria pertaining to public disruption, awareness and perception were also excluded. Although the professional groups acknowledged disease prioritization was often driven by public and political pressure, they agreed it should not steer the process [7]. The final 21 selected criteria could all be quantitatively measured with scientific information available in the literature.

**Table 2:** Disease criteria and standardized part-worth utility values (β) for disease criteria levels by country.

Disease criteria^1^ and corresponding levels	Canada		US		*t* 4
	*MUV* 2	*SD* 3	*MUV* 2	*SD* 3	
*Duration of illness in humans*					
No illness observed or only a few days of illness	−47.58	22.40	−46.58	31.05	0.72∧
Short-term illness (weeks)	−26.00	23.78	−22.08	16.16	3.78*** ∧
Medium-term illness (months)	6.26	21.34	3.24	23.55	2.64** ∧
Chronic illness (years) or illness with permanent deficits	67.32	30.94	65.42	30.09	1.23
*Transmission potential from animals to humans*					
No transmission from animals to humans	−38.81	18.86	−41.89	17.39	3.33** ∧
Low transmission from animals to humans	−28.33	18.40	−33.07	17.43	5.19***
Moderate transmission from animals to humans	5.23	17.15	13.03	16.83	9.00***
High	61.91	21.12	61.93	21.09	0.02
*Disease trend in Canada/US in the last five years in animals*					
Decline over the last five years	−47.50	21.98	−48.38	21.66	0.80
Stable over the last five years	−24.50	18.50	−26.24	19.01	1.82
Increase over the last five years	25.03	18.23	28.13	17.74	3.38**
New emerging disease, rapid increase over the last five years	46.97	22.54	46.49	24.71	0.40∧
*Economic burden in humans*					
No cost to the health care system and individuals	−35.66	26.38	−34.54	24.26	0.86∧
Low cost ($100 per sick individual)	−14.78	22.27	−22.16	18.03	7.14*** ∧
Moderate cost ($1,000 per sick individual)	6.50	16.63	8.61	16.44	2.50*
High cost ($10,000 per sick individual)	43.94	36.21	48.10	31.32	2.41* ∧
*Transmission potential from humans to animals*					
No transmission from humans to animals	−26.88	17.37	−27.19	21.58	0.31∧
Low transmission from humans to animals	−25.98	18.96	−27.03	19.44	1.07
Moderate transmission from humans to animals	6.74	15.47	13.44	15.48	8.49***
High transmission from humans to animals	46.13	26.65	40.79	22.95	4.21*** ∧
*Duration of illness in animals*					
No illness observed or only a few days of illness	−28.32	19.05	−33.47	18.99	5.31***
Short-term illness (weeks)	−9.97	20.26	−10.85	17.58	0.91∧
Medium-term illness (months)	1.21	18.71	6.68	17.37	5.93*** ∧
Chronic illness (years) or illness with permanent deficits	37.08	24.28	37.63	22.53	0.47∧
*Transmission potential between animals*					
No transmission between animals	−27.27	21.02	−23.24	18.21	4.01*** ∧
Low transmission between animals	−13.53	15.78	−18.35	13.60	6.42*** ∧
Moderate transmission between animals	4.76	15.33	3.52	18.29	1.45∧
High transmission between animals	36.03	18.62	38.08	18.44	2.17*

1 Presented in order of importance to Canadian participants.

2 Mean part-worth utility values (β) across respondents.

3 Standard deviation of mean part-worth utility values (β) across respondents.

4 *t*-statistic; *d.f*. = 1,537.

* *p*<0.05,

** *p*<0.01,

*** *p*<0.001.

∧ Adjusted for unequal variance using the Welch *t*-test; Satterthwaite’s *d.f.* = 1,282.61 to 1531.22.

### Disease Selection and Literature Search

We identified 62 existing and emerging zoonotic and enteric diseases of public health importance in North America (Tables 4, 5). These diseases were selected using the following criteria: a) nationally notifiable to the Public Health Agency of Canada (PHAC), Canadian Food Inspection Agency (CFIA), Centers for Disease Control and Prevention (CDC) or the United States Department of Agriculture (USDA), b) internationally notifiable to the World Health Organization (WHO), the World Organization for Animal Health (OIE) or the Food and Agriculture Organization (FAO) or c) identified as a priority by PHAC at a national meeting on non-enteric zoonoses in 2009. We note that the majority of enteric diseases in this study are zoonotic; hence, the group of diseases in this paper will be referred to as zoonotic diseases. However, there are three diseases in this study that are strictly enteric diseases with no zoonotic involvement, these are, Cholera, Hepatitis A and Paralytic Shellfish Poisoning (PSP). As enteric diseases typically fall under the responsibility of the Zoonotic Division in public health (for example, PHAC and the CDC), these diseases were also included in our study.

**Table 3:** Disease criteria and standardized part-worth utility values (β) for disease criteria levels by country.

Disease criteria^1^ and corresponding levels	Canada		US		*t* 4
	*MUV* 2	*SD* 3	*MUV* 2	*SD* 3	
*Economic and social burden on trade in animals*					
No cost to trade in animals	−25.21	16.44	−15.51	19.10	10.69*** ∧
Low cost to trade in animals (vaccination of herds)	−11.08	17.47	−16.85	19.54	6.10*** ∧
Moderate cost to trade in animals (restriction of movement and trade)	1.26	12.90	2.33	17.34	1.38∧
High cost to trade in animals (culling of herds or destroying infected crops/produce)	35.03	23.65	30.02	21.60	4.34*** ∧
*Severity of illness in animals*					
No apparent clinical signs or the animal-source of infection is non-living (e.g. food-source)	−20.92	18.56	−24.17	15.23	3.75*** ∧
Mild clinical signs (minor distress in animals such as fever, lethargy, shivering, constipation, loose feces)	−15.40	16.98	−13.87	16.75	1.78
Moderate clinical signs (moderate distress in animals such as difficult breathing, bleeding from openings,aborted fetuses)	4.75	18.39	4.41	20.16	0.35∧
Severe clinical signs (severe distress in animals such as convulsion, organ failure, neurological involvement)	31.57	19.80	33.64	18.07	2.14* ∧
*High risk groups in humans*					
No	−25.66	18.68	−24.05	14.53	1.89∧
Yes	30.39	20.31	29.32	17.19	1.12∧
Unknown	−4.73	16.74	−5.28	14.27	0.69∧
*Control measures in humans*					
Highly effective in reducing disease burden	5.53	33.46	3.75	32.58	1.06
Moderately effective in reducing disease burden	−0.52	18.55	−7.72	17.88	7.76***
Minimally effective in reducing disease burden	−5.00	17.71	0.52	19.35	5.84*** ∧
Not effective at all in reducing disease burden	−0.01	31.90	3.46	27.23	2.29* ∧
*Control measures in animals*					
Highly effective in reducing disease burden	9.02	27.81	5.17	23.03	2.96** ∧
Moderately effective in reducing disease burden	1.34	18.40	−2.47	15.26	4.42*** ∧
Minimally effective in reducing disease burden	−6.21	19.71	−0.66	15.35	6.16*** ∧
Not effective at all in reducing disease burden	−4.15	26.50	−2.04	22.38	1.68∧
*How much is known scientifically about the disease*					
Knowledge of the disease is well known and scientifically valid	−10.17	27.16	−3.96	30.17	4.26*** ∧
Knowledge of the disease exists but the validity of the information is uncertain	−2.97	23.19	4.23	16.08	7.06*** ∧
Knowledge of the disease is currently insufficient	6.91	17.24	3.39	23.08	3.39** ∧
There is no scientific knowledge of the disease	6.22	18.96	−3.66	18.58	10.33***
*High risk groups in animals*					
No	−13.79	17.69	−13.37	13.23	0.53∧
Yes	14.40	16.49	13.73	18.63	0.75∧
Unknown	−0.61	14.64	−0.36	15.22	0.33

1 Presented in order of importance to Canadian participants.

2 Mean part-worth utility values (β) across respondents.

3 Standard deviation of mean part-worth utility values (β) across respondents.

4 *t*-statistic; *d.f*. = 1,537.

* *p*<0.05,

** *p*<0.01,

*** *p*<0.001.

∧ Adjusted for unequal variance using the Welch *t*-test; Satterthwaite’s *d.f.* = 1,282.61 to 1531.22.

A literature search was conducted for each zoonosis in the study. Diseases exhibiting multiple forms (e.g. acute/chronic, latent/active, encephalitic/non-encephalitic) were divided into separate syndromes and assigned approximate proportions that were informed by the literature. There were 117 separate disease syndromes identified from the 62 diseases (Tables 4, 5). The literature search obtained information for each criterion for each disease syndrome. The literature search included: a) website searches of human and animal health organizations involved in zoonotic disease prevention and control including national organizations, intergovernmental organizations, provincial organizations and academic institutions; b) reference textbooks [2], [28], [29] and c) PubMed cataloged peer-reviewed publications. Key search terms used included the disease criterion, the scientific and/or common name of diseases, and a combination of the two (e.g. *case-fatality rate* and/or *rabies*).

**Table 4:** List of 62 diseases and their separate disease syndromes and proportions.

#	Diseases or disease syndromes	Proportion	#	Diseases or disease syndromes	Proportion
**1**	1. American Trypanosomiasis/Chagas' disease (acute)	70%	**31**	17. Cyclosporiasis (healthy individuals)	98%
**2**	1. American Trypanosomiasis/Chagas' disease (chronic)	30%	**32**	17. Cyclosporiasis (immunocompromised individuals)	2%
**3**	2. Anaplasmosis/Canine granulocytic anaplasmosis (acute)	50%	**33**	18. Cysticercosis/Taeniasis (non-neurological involvement)	99.8%
**4**	2. Anaplasmosis/Canine granulocytic anaplasmosis(chronic)	50%	**34**	18. Cysticercosis/Taeniasis (neurocysticercosis)	0.2%
**5**	3. Anthrax (cutaneous)	95%	**35**	19. Dengue fever	99%
**6**	3. Anthrax (inhalational)	5%	**36**	19. Dengue haemorrhagic fever	1%
**7**	4. Babesiosis (mild)	95%	**37**	20. Eastern equine encephalitis (non-neurological involvement)	66.7%
**8**	4. Babesiosis (shock and renal failure)	5%	**38**	20. Eastern equine encephalitis (encephalitic/neurological involvement)	33.3%
**9**	5. Bartonellosis (Cat-scratch disease) (mild)	90%	**39**	21. Ebola virus haemorrhagic fever (*Reston* strain)	0.4%
**10**	5. Bartonellosis (Cat-scratch disease) (bacteremia/systemic disease)	10%	**40**	21. Ebola virus haemorrhagic fever (other Ebola strains)	99.6%
**11**	6. Baylisascariasis (visceral larval migrans)	95%	**41**	22. Echinococcosis (cystic)	70%
**12**	6. Baylisascariasis (neural or ocular larval migrans)	5%	**42**	22. Echinococcosis (alveolar)	30%
**13**	7. Botulism (mild)	50%	**43**	23. *Escherichia coli* infection	85%
**14**	7. Botulism (paralysis)	50%	**44**	23. *Escherichia coli* infection (hemolytic-uremic syndrome)	15%
**15**	8. Bovine Tuberculosis (latent)	92.5%	**45**	24. Giardiasis (healthy individuals)	98%
**16**	8. Bovine Tuberculosis (active)	7.5%	**46**	24. Giardiasis (immunocompromised individuals)	2%
**17**	9. Brucellosis (mild)	75%	**47**	25. Hantavirus pulmonary syndrome (moderate)	50%
**18**	9. Brucellosis (undulant)	25%	**48**	25. Hantavirus pulmonary syndrome (respiratory failure)	50%
**19**	10. Campylobacteriosis (healthy individuals)	98%	**49**	26. Hendra virus (rare, consistent prognosis suspected)	100%
**20**	10. Campylobacteriosis (immunocompromised individuals)	2%	**50**	27. Hepatitis A (mild)	85%
**21**	11. Chlamydiosis (*C. abortus* spp.)	50%	**51**	27. Hepatitis A (prolonged relapse)	15%
**22**	11. Chlamydiosis (*C. felis* spp.)	50%	**52**	28. H1N1 Influenza (mild)	97.5%
**23**	12. Cholera (mild)	80%	**53**	28. H1N1 Influenza (respiratory failure)	2.5%
**24**	12. Cholera (severe dehydration, kidney failure,hypovolemic shock)	20%	**54**	29. HPAI H5N1 Influenza (moderate)	40%
**25**	13. Coccidioidomycosis (acute)	99%	**55**	29. HPAI H5N1 Influenza (respiratory failure)	60%
**26**	13. Coccidioidomycosis (disseminated)	1%	**56**	30. Japanese encephalitis (mild)	99.5%
**27**	14. Crimean-Congo haemorrhagic fever (consistent prognosis)	100%	**57**	30. Japanese encephalitis (neurological involvement)	0.5%
**28**	15. Cryptosporidiosis (healthy individuals)	98%	**58**	31. La Cross virus (non-encephalitic)	99%
**29**	15. Cryptosporidiosis (immunocompromised individuals)	2%	**59**	31. La Cross virus (encephalitic)	1%
**30**	16. Cutaneous larva migrans/Ancylostomiasis (dose-dependent)	100%			

When there was conflicting information between multiple sources, the most consistent sources were used to define the disease criteria; where only two sources were available, the most recent source was used. When information was not available for North America, information available for other developed countries was used. Country-specific information for Canada and the US were collected for four criteria – *disease incidence in the last five years* (humans and animals) and *disease trend in the last five years* (humans and animals). An assumption was made that the remaining criteria were consistent between countries. When there was a gap in the literature, particularly pertaining to animal-related criteria [30], information from corresponding human-related criteria were used to define animal-related criteria on the assumption that disease criteria were similar between humans and animals. Animal-related criteria, however, were not used to inform human-related criteria due to existing data for humans.

**Table 5:** List of 62 diseases and their separate disease syndromes and proportions.

#	Diseases or disease syndromes	Proportion	#	Diseases or disease syndromes	Proportion
**60**	32. Lassa fever (mild)	80%	**89**	47. Rift Valley fever (mild)	99%
**61**	32. Lassa fever (severe multi-system failure)	20%	**90**	47. Rift Valley fever (haemorrhagic fever)	1%
**62**	33. Leishmaniasis (mild cutaneous)	30%	**91**	48. Rocky Mountain spotted fever (US only, prognosis unknown)	100%
**63**	33. Leishmaniasis (moderate cutaneous)	33.7%	**92**	49. Salmonellosis (enteric)	98%
**64**	33. Leishmaniasis (visceral (kala-azar))	33.3%	**93**	49. Salmonellosis (septicemic/enteric in immunocompromised)	2%
**65**	34. Leptospirosis (aniceteric)	93%	**94**	50. Severe Acquired Respiratory Syndrome (mild)	90%
**66**	34. Leptospirosis (icteric)	7%	**95**	50. Severe Acquired Respiratory Syndrome (respiratory failure)	10%
**67**	35. Listeriosis (healthy individuals)	50%	**96**	51. Shigellosis (mild)	95%
**63**	35. Listeriosis (pregnant, newborns, elderly, immunocompromised)	50%	**97**	51. Shigellosis Reiter's (syndrome and chronic arthritis)	5%
**69**	36. Lyme disease (early localized)	50%	**98**	52. St. Louis encephalitis (non-encephalitic)	99%
**70**	36. Lyme disease (early dissemination)	49%	**99**	52. St. Louis encephalitis (encephalitic)	1%
**71**	36. Lyme disease (chronic dissemination)	1%	**100**	53. Toxocariasis (visceral larval migrans)	98%
**72**	37. Marburg haemorrhagic fever (acute)	80%	**101**	53. Toxocariasis (ocular larval migrans)	2%
**73**	37. Marburg haemorrhagic fever (chronic)	20%	**102**	54. Toxoplasmosis (healthy individuals)	95%
**74**	38. Monkeypox (consistent prognosis)	100%	**103**	54. Toxoplasmosis (immunocompromised individuals)	5%
**75**	39. Nipah virus encephalitis (acute)	80%	**104**	55. Trichinosis (dose-dependent)	100%
**76**	39. Nipah virus encephalitis (residualneurological deficits)	20%	**105**	56. Tularemia (ulceroglandular and glandular)	90%
**77**	40. Old/New World Screwworm (consistent prognosis)	100%	**106**	56. Tularemia (typhoidal)	10%
**78**	41. Paralytic shellfish poisoning (dose-dependent)	100%	**107**	57. Typhus fever (epidemic louse-borne)	50%
**79**	42. Plague (bubonic)	90%	**108**	57. Typhus fever (endemic flea-borne)	50%
**80**	42. Plague (septicemic)	7.5%	**109**	58. variant Creutzfeldt-Jakob disease (CJD)/BSE (consistent prognosis)	100%
**81**	42. Plague (pneumonic)	2.5%	**110**	59. Venezuelan equine encephalitis (non-encephalitic)	97.5%
**82**	43. Powassan virus (moderate leading to recovery)	66.7%	**111**	59. Venezuelan equine encephalitis (encephalitic)	2.5%
**83**	43. Powassan virus (severe leading to death)	33.3%	**112**	60. West Nile virus (non-neurological involvement)	80%
**84**	44. Psittacosis/Avian Chlamydiosis (mild)	99%	**113**	60. West Nile virus (neurological involvement)	20%
**85**	44. Psittacosis/Avian Chlamydiosis (severe multi-system failure)	1%	**114**	61. Western equine encephalitis (systemic, non-neurological involvement)	96.5%
**86**	45. Q fever (acute)	90%	**115**	61. Western equine encephalitis (encephalitic, neurological involvement)	3.5%
**87**	45. Q fever (chronic)	10%	**116**	62. Yellow fever (mild)	85%
**88**	46. Rabies (consistent prognosis)	100%	**117**	62. Yellow fever (hepato-renal failure)	15%

### Defining Levels for Disease Criteria

Three or four levels for each criterion were defined according to the range exhibited in the literature (Tables 1, 2, 3). For example, the duration of illness in humans among diseases ranged from no duration (asymptomatic) to chronic disability (permanent deficits). For the criteria, *case-fatality* and *disease incidence* (both humans and animals), levels were defined according to the 25^th^, 50^th^, 75^th^ and 90^th^ percentile values.

### Survey Development and Administration

**Figure 1:**
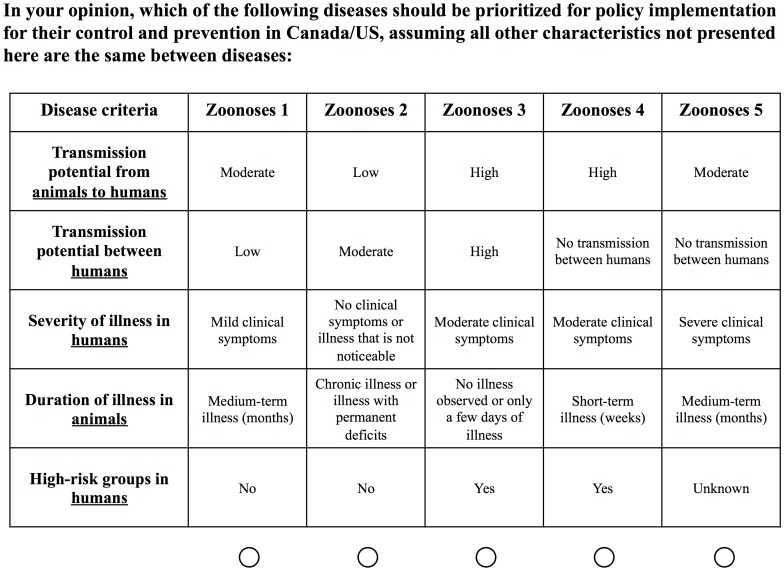
Example of one choice task set completed by each study participant. As multiple survey versions were administered randomly to each person, a different combination of disease criteria and levels was presented to study participants. The ordering of the presentation of disease criteria within each choice task was randomized to reduce ordering bias.

We used 21 criteria with criteria levels ranging from three to four levels that reflected the full range of each criterion across the 62 diseases to inform our survey instrument. Due to the large number of criteria, a partial-profile choice-based conjoint (CBC) survey was developed comprising of 14 choice tasks [31], [32]; each choice task presented participants with five disease combinations (zoonoses 1–5) described by criteria levels for 5 of the 21 criteria. Disease criteria and levels varied between choice tasks. Participants were asked to select one zoonosis to prioritize in either Canada or the US with the objective being for policy implementation for control and prevention (Figure 1). Surveys were administered electronically to allow for flexibility in the study design and wide participation across North America. Surveys were offered in English, French and Spanish. We used Sawtooth Software CBC module v7 to create 300 survey versions to generate an efficient experimental design (*D*-efficiency of 908.13326 relative to a full-orthogonal design with a standard error of <0.05 for each criterion level). The selected design ensured a number of important features of an efficient experimental design were included, these were: level balance (each criteria level appeared approximately an equal number of times across the 14 choice tasks), orthogonality (criteria levels were selected independently of other criteria levels) and a balanced overlap approach (the design permitted levels to appear more than once per choice task for a more precise measurement of interaction terms) [33]. Disease combinations were designed to be easily understood, to mimic the presentation of zoonoses in nature and to enhance informant and statistical efficiency [24], [31], [32], [34].

Two additional fixed choice tasks were included in all survey versions to determine the reliability of responses. In these choice tasks, one zoonosis was more severe as described by the set of criteria compared to the remaining four, giving participants an incentive to select that zoonosis to prioritize. Fixed choice task 1 presented one zoonosis with the highest incidence in humans (10,000 cases), most severe illness in humans (severe clinical symptoms), highest transmission potential between humans (high), highest case-fatality in humans (80%) and the most costly economic burden in humans ($10,000 per sick individual). In comparison, the remaining four zoonoses contained a combination of lower and less severe criteria levels. Fixed choice task 2 presented one zoonosis with the most severe illness in animals (severe clinical symptoms), highest case-fatality in animals (80%), most costly socioeconomic burden in trade in animals (high cost such as culling of herds or destroying infected crops/produce), longest duration of illness in animals (chronic illness or permanent deficits) and rapid change in disease trend in the human population (new emerging disease, rapid increase over the last five years). In comparison, the remaining four zoonoses contained a combination of lower and less severe criteria levels. The fixed choice tasks tested the reliability of responses by identifying respondents who did not understand the choice task process and/or fatigue responders. The ordering of the presentation of disease criteria within each choice task was randomized to reduce ordering bias. The fixed choice tasks were also randomized to reduce ordering bias and as an additional measure of reliability given the tasks were designed to test the reliability of respondents to understand the choice task process rather than the reliability of respondents to make the same choices consistently across the two fixed choice task sets. Sawtooth Software SSI Web v7 was used to randomly assign a survey version to each study participant.

### Study Participants

The study was approved by the Research Ethics Board at the University of Guelph. The target study participants were individuals from the public in Canada and the US with no knowledge or experience in the prioritization of zoonotic diseases. Participants from both countries were recruited using an online panel through Research Now™; these are groups of pre-screened individuals who have expressed a willingness to participate in online surveys. Surveys were collected anonymously. All participants acknowledged an informed consent assuring confidentiality and the option to withdraw from participation without penalty. Sawtooth Software SSI Web v7 was used to screen participants through a series of demographic questions prior to survey commencement. Participants were disqualified if they did not reside in North America or were employed in the following fields: epidemiology, public health, medical sciences, veterinary sciences, infectious disease research, laboratory technician, nursing or dentistry. Quotas were used to ensure that no particular age group, gender or geographic region dominated the survey responses and that study populations were representative of the national populations in their respective countries (Table 6).

**Table 6:** Demographic characteristics of Canadian and US study participants in comparison to their respective national population characteristics.

	Canada (n = 761)			US (n = 778)				
	Study Participant	National Population^1^	χ^2^		Study Participant		National Population^2^	χ^2^
**Gender** *Male*	48.0%	48.5%	0.09	**Gender**	48.2%		49.2%	0.31
*Female*	52.0%	51.5%			51.8%		50.8%	
**Age group** *18 to 34*	27.3%	27.9%	14.92*	**Age group**	29.7%		30.6%	0.30
*35 to 50*	35.1%	29.1%			27.5%		27.2%	
*50+*	37.5%	43.0%			42.8%		42.2%	
*Unknown*	0.1%	–			–		–	
**Province** *Alberta*	10.6%	10.6%	12.9	**Region** 3				0.64
*British Columbia*	13.1%	13.4%		*Midwest*	22.6%		21.7%	
*Manitoba*	3.8%	3.5%		*Northeast*	18.1%		18.3%	
*New Brunswick*	2.1%	2.3%		*South*	35.9%		37.0%	
*Newfoundland and Labrador*	1.4%	1.6%		*West*	23.4%		23.0%	
*Nova Scotia*	2.8%	2.8%						
*Northwest Territories*	0.1%	0.1%						
*Nunavut*	0.0%	0.1%						
*Ontario*	38.9%	38.2%						
*Prince Edward Island*	0.9%	0.4%						
*Quebec*	22.7%	23.9%						
*Saskatchewan*	3.0%	3.0%						
*Yukon*	0.4%	0.1%						
**Educational attainment** 4				**Educational attainment** 5				
*High school graduate or less*	34.8%	45.1%	262.39*			42.9%	44.5%	307.07*
*Diploma, trade or college degree*	25.4%	35.1%				4.5%	27.0%	
*Bachelor's degree*	27.1%	12.7%				35.0%	18.7%	
*Master's degree*	7.4%	5.8%				13.0%	7.1%	
*Professional degree (MD, DVM)*	3.3%	0.6%				2.8%	1.4%	
*Doctorate degree*	1.5%	0.8%				1.8%	1.3%	
*Unknown*	0.7%	–				–	–	

1 2011 population data for individuals 18 years and older in Canada was obtained from Statistics Canada [36].

2 2010 population data for individuals 18 years and older in the US was obtained from the US Census Bureau [38].

3 Regions were: **Midwest** (Illinois, Indiana, Iowa, Kansas, Michigan, Minnesota, Missouri, Nebraska, North Dakota, Ohio, South Dakota, Wisconsin); **Northeast** (Connecticut, Maine, Massachusetts, New Hampshire, New Jersey, New York, Pennsylvania, Rhode Island, Vermont); **South** (Alabama, Arkansas, Delaware, District of Columbia, Florida, Georgia, Kentucky, Louisiana, Maryland, Mississippi, North Carolina, Oklahoma, South Carolina, Tennessee, Texas, Virginia, West Virginia); **West** (Alaska, Arizona, California, Colorado, Hawaii, Idaho, Montana, Nevada, New Mexico, Oregon, Utah, Washington, Wyoming).

4 2006 education data for individuals 20 years and over in Canada (most current and available data) [35].

5 2010 education data for individuals 18 years and over in the US [37].

* *p*<0.001.

### Data Analysis

We used Chi Square and Mann-Whitney tests to compare the demographic and survey characteristics of study participants to their respective national populations and to make comparisons by country. National population data for age, gender, education and region was obtained from Statistics Canada [35], [36] and the US Census Bureau [37], [38]. Unpaired t-tests and *F*-tests were used to explore differences in standardized importance scores and part-worth utility values between Canada and the US. Twenty-one part-worth utility values, one for each disease criterion, were assigned to the 117 separate disease syndromes by matching the level of each disease criterion to those of disease syndromes. Part-worth utility values were summed up in proportion to the relative frequency of each syndrome within a disease (Tables 4, 5) to derive an overall score for each of the 62 diseases. The overall scores were used to rank-order diseases; the higher the score, the higher the ranking on the priority list. We chose to use summed part-worth utility values over other approaches such as market simulations because we wanted to apply CA-derived scores to a set of disease using a method that is comparable to current traditional prioritization methods [4], [11]–[13], [16]–[18]. We also selected this approach because our goal was to prioritize the list of diseases in ranked order, which can be achieved using the simple summed part-worth utility values approach.

Hierarchical Bayes (HB) was used to compute parameter estimates (weighted scores) from survey choice data [39]. We used Sawtooth Software CBC/HB v5.2.8 to estimate individual-level parameter estimates (β). The software uses a combination of Bayes theorem, a Monte Carlo Markov Chain (MCMC) procedure and the Metropolis/Hastings algorithm to derive parameter estimates from two distributions: an upper-level model (prior) drawn from a multivariate normal distribution representing parameter estimates at the population level and a lower-level model (posterior) described by a multinomial logit model representing parameter estimates at the individual level. Bayesian updating of probabilities using MCMC and the Metropolis/Hastings algorithm provided an iterative process to update parameter estimates drawing on the upper-level and lower-level model [39]. The final individual-level parameter estimates reflected an optimal mix of the population average and individual choices [40]. We computed 30,000 preliminary iterations before convergence was assumed (and observed) and an additional 30,000 iterations per respondent to estimate parameters.

Zero-centered part-worth utility values (β estimates) were standardized by setting the average range of the parameter values of all disease criteria to 100. Part-worth utility values represent the relative influence each criterion level had on respondent choices with higher values indicating a stronger influence on choice [24]. To estimate the influence of each criterion collectively, importance scores were calculated as a percentage by dividing the difference in range between the highest and lowest criterion level part-worth utility value by the sum of all part-worth utility value ranges across all criteria. The more variation between the levels in a criterion, the higher the importance score and the stronger the influence the criterion had on the decision to prioritize [24]. Part-worth utility values and importance scores were calculated directly using Sawtooth Software SMRT v4.22.0. We computed *t*-statistics by dividing the mean difference in range in part-worth utility values across each criterion by the standard error of the differences to test each disease criterion for statistical significance in the final model. The goodness of fit of the individual-level HB models were estimated using Sawtooth Software CBC/HB v5.2.8 and are presented as a percent certainty fit and a root likelihood (RLH); both of these measures are calculated as the probability of each respondent choosing as he/she did on each choice task using a logit model fitted with the current estimates of each respondent’s part-worth utility values [39]. The percent certainty and RLH both indicate how much better the model is than a chance model, as compared to a perfect model.

## Results

### Demographic and Survey Characteristics

In total, 46,547 and 8,298 email invitations were sent to Canadian and US participants, respectively. Of these, 1,313 (2.8%) and 1,309 (15.8%) completed surveys were returned in 8 and 7 business days, respectively. The majority of completed surveys were in English (77.1% in Canada, 99.4% in the US) while remaining surveys were completed in French (22.9% in Canada) and Spanish (0.6% in US). Participants passed the survey if all 14 choice task sets were completed and the correct diseases were selected for both fixed choice tasks^2^. The pass rate was 58.0% in Canada (761) and 59.4% in the US (778); there was no significant difference in the pass rate between countries (χ^2^ = 0.59, *p* = 0.44). The median completion time for passed surveys was 26.9 minutes in Canada and 28.1 minutes in the US. There were 1,539 completed and passed surveys in this study.

The study population was generally representative of the national population by gender, age and geography (Table 6). There was a significantly higher response rate in the 35 to 50 years age group in the Canadian study population compared to the Canadian national population (*p*<0.001). A higher than expected educated population was observed in both study populations compared to their respective national populations (*p*<0.001 for both countries).

### Model Fit

Both the Canadian and the US models had a percent certainty fit of 79.4% and a root likelihood (RLH) of 0.72. The expected percent certainty for a chance model is 0% and a perfect model is 100% while the expected RLH for a chance model is 0.2 (one divided by five disease combinations per task) and a perfect model is 1.0 [39].

### Disease Criteria Importance Scores and Part-worth Utility Values

The importance scores for disease criteria indicate the degree to which each criterion contributed to the decision to prioritize (Table 7). Human-related criteria were preferred over corresponding animal-related criteria with each of the eight matching criteria exhibiting this trend in both countries. The four transmission potential criteria were ranked in the following order of preference in both countries: human-human, animal-human, humans-animals and animal-animal; thus also revealing a stronger preference for human-related criteria over animal-related criteria. Although the contribution of each disease criterion in the decision to prioritize zoonoses varied, each criterion was statistically significant (*P*-value <0.05) in the final model for both countries validating the choice of appropriate criteria for assessing disease prioritization and highlighting their varying degree of importance in the overall decision.

**Table 7:** Disease criteria importance scores by country.

Disease criteria	Canada (n = 761)			US (n = 778)			*t* 6
	*R* 3	*MS* 4	*SD* 5	*R*	*MS*	*SD*	
Case-fatality (H)^1^	1	8.57	2.20	1	**9.84**	2.04	11.81*** ∧
Incidence of the disease in the last five years (H)	2	8.18	2.44	2	**9.06**	2.31	7.29***
Case-fatality (A)^2^	3	**6.91**	2.06	5	6.16	1.48	8.17*** ∧
Incidence of the disease in the last five years (A)	4	6.63	2.20	3	**7.03**	1.70	3.98*** ∧
Severity of disease (H)	5	6.41	1.88	4	6.39	1.95	0.19
Disease trend in the last five years (H)	6	**6.16**	1.70	7	5.81	1.69	4.14***
Transmission potential between humans	7	**6.16**	1.44	8	5.59	1.50	7.59***
Duration of illness (H)	8	5.82	1.95	6	5.85	1.83	0.29
Transmission potential from animals to humans	9	5.15	1.41	9	5.27	1.38	1.81
Disease trend in the last five years (A)	10	4.89	1.53	10	4.99	1.50	1.35
Economic burden (H)	11	4.43	2.08	11	4.51	1.85	0.79∧
Transmission potential from humans to animals	12	4.01	1.75	12	4.01	1.40	0.50∧
Duration of illness (A)	13	3.72	1.14	13	3.76	1.34	0.64∧
Transmission potential between animals	14	3.48	1.24	14	3.39	1.23	1.37
Economic and social burden on trade (A)	15	**3.29**	1.33	16	3.10	1.23	2.93** ∧
Severity of disease (A)	16	3.15	1.19	15	3.24	1.10	1.62∧
High risk groups (H)	17	**3.05**	1.32	17	2.76	1.16	4.54*** ∧
Control measures (H)	18	**2.87**	1.63	18	2.71	1.46	1.98* ∧
Control measures (A)	19	**2.61**	1.37	20	2.12	1.14	7.54*** ∧
How much is known scientifically about the disease	20	2.57	1.20	19	2.51	1.32	0.92∧
High risk groups (A)	21	1.95	1.07	21	1.87	1.03	1.37

1 (H) = human-related characteristic, for example, case-fatality in *humans*.

2 (A) = animal-related characteristic, for example, case-fatality in *animals*.

3 Relative rank of disease criteria by importance scores; presented in order of importance to Canadian participants.

4 Mean importance score across respondents.

5 Standard deviation of importance scores across respondents.

6 *t*-statistic; *d.f*. = 1,537.

* *p*<0.05.

** *p*<0.01.

*** *p*<0.001.

∧ Adjusted for unequal variance using the Welch *t*-test; Satterthwaite’s *d.f.* = 1,377.25 to 1529.68.

Scores in **bold** indicate disease criteria with statistically significant difference in importance scores between Canada and the US; scores for the country with the highest score (i.e. placed more importance on) are in **bold**.

The part-worth utility values (β) indicate the relative influence each level had on respondent choices with higher values representing a stronger degree of influence on choice (Tables 1, 2, 3). The part-worth utility value trends for the ten disease criteria with significant difference in importance score between countries (Table 7) can be broadly summarized as follows:

Both groups considered *case-fatality in humans* and *incidence of the disease in the last five years in humans* to be the most influential criteria in the decision to prioritize zoonoses (Table 7). Although the US group was influenced more strongly by these criteria (*P*-value <0.001 for both, Table 7), expressed by a wider range in part-worth utility values between the lowest and highest levels (Tables 1, 2, 3), there was agreement between countries on the importance placed on the levels within these criteria. The same trend was observed for the criterion *incidence in the last five years in animals*.

The Canadian group was more strongly influenced by case-fatality in animals, disease trend in the last five years in humans, transmission potential between humans, economic and social burden on trade in animals and high risk groups in humans (P-value <0.01 for all, Table 7), nonetheless, there was agreement between countries on the levels of least importance (lowest part-worth utility values) and levels of highest importance (highest part-worth utility values) with incremental increases in part-worth utility values for the levels in between (Tables 1, 2, 3).

The criteria *control measures in humans* and *control measures in animal* did not exhibit this sequential pattern in level preference in either country, however, these criteria were statistically significant (*P*-value <0.05) in the final models. Although preference was given to ‘highly effective in reducing disease burden’ (highest part-worth utility values) in both humans and animals and in both countries, there was no clear order of preference in the remaining levels (Tables 1, 2, 3). Both groups expressed next preference for the level ‘not effective at all in reducing disease burden’ in humans (second highest part-worth utility values). While preferences appear contradictory, this is due to the difference in preferences between individuals who choose to prioritize when the opportunity to control is attainable (highly effective control measures) and those who choose to prioritize when the opportunity to control is not viable (no control measures).

Although the strength of preference in disease criteria importance scores differed between countries, there was general agreement in the contribution of disease criteria in the decision to prioritize zoonoses (Table 7). However, marginal differences in the part-worth utility values within disease criteria (Tables 1, 2, 3) and in disease incidence and trend contributed to a unique disease priority list by country (Table 8).

**Table 8:** Disease priority list by country.

Canada	Score	rank	US	score	rank	Difference in rank (relative to Canada)
Nipah virus encephalitis	284.01	1	variant Creutzfeldt-Jakob disease (CJD)	368.89	1	5
Rabies	280.02	2	Rabies	295.44	2	0
Ebola virus haemorrhagic fever	260.24	3	Nipah virus encephalitis	286.10	3	−2
Marburg haemorrhagic fever	225.13	4	Ebola virus haemorrhagic fever	276.87	4	−1
Influenza (H1N1)	208.70	5	Marburg haemorrhagic fever	250.86	5	−1
variant Creutzfeldt-Jakob disease (CJD)	194.02	6	Influenza (H1N1)	207.22	6	−1
Listeriosis	177.78	7	Listeriosis	200.75	7	0
Hendra virus	64.79	8	Tularemia	164.88	8	4
Influenza (H5N1)	64.69	9	Anaplasmosis/Canine granulocytic anaplasmosis	137.19	9	36*
Salmonellosis	37.65	10	Hantavirus pulmonary syndrome	106.09	10	10*
Leishmaniasis	23.44	11	Paralytic shellfish poisoning	104.85	11	25*
Tularemia	10.33	12	Babesiosis	90.74	12	38*
Escherichia coli infection	−8.46	13	American trypanosomiasis (Chagas' disease)	81.17	13	5
Cryptosporidiosis	−11.29	14	Plague	79.65	14	9
Eastern Equine Encephalitis	−26.50	15	Hendra virus	65.12	15	−7
Botulism	−33.51	16	Influenza (H5N1)	62.25	16	−7
Shigellosis	−36.76	17	Shigellosis	55.89	17	0
American trypanosomiasis (Chagas' disease)	−52.78	18	Eastern Equine Encephalitis	54.28	18	−3
Giardiasis	−54.12	19	Leishmaniasis	53.60	19	−8
Hantavirus pulmonary syndrome	−59.94	20	Salmonellosis	47.74	20	−10*
Campylobacteriosis	−60.02	21	Escherichia coli infection	38.07	21	−8
Toxoplasmosis	−60.58	22	Q fever	19.95	22	5
Plague	−62.54	23	Cryptosporidiosis	10.44	23	−9
Psittacosis/Avian chlamydiosis	−74.75	24	Rocky Mountain spotted fever	7.94	24	9
Leptospirosis	−79.55	25	Botulism	−26.23	25	−9
Chlamydiosis	−79.67	26	Campylobacteriosis	−27.72	26	−5
Q fever	−94.88	27	Leptospirosis	−32.95	27	−2
West Nile virus	−109.20	28	Lyme Disease	−45.26	28	2
Bartonellosis	−114.43	29	Brucellosis	−47.38	29	5
Lyme Disease	−124.52	30	Chlamydiosis	−52.67	30	−4
Crimean-Congo haemorrhagic fever	−130.11	31	Psittacosis/Avian chlamydiosis	−53.33	31	−7
Powassan virus	−142.24	32	Toxoplasmosis	−58.94	32	−10*
Rocky Mountain spotted fever	−145.83	33	Giardiasis	−70.07	33	−14*
Brucellosis	−149.39	34	Powassan virus	−84.47	34	−2
Anthrax	−167.66	35	West Nile virus	−85.51	35	−7
Paralytic shellfish poisoning	−170.06	36	Bartonellosis	−94.16	36	−7
Echinococcosis	−180.88	37	Typhus	−103.02	37	9
Toxocariasis	−183.97	38	Coccidioidomycosis	−109.79	38	20*
Cutaneous larva migrans	−199.71	39	Crimean-Congo haemorrhagic fever	−132.98	39	−8
Lassa fever	−203.02	40	Anthrax	−144.16	40	−5
Baylisascariasis	−219.41	41	Echinococcosis	−147.03	41	−4
Severe Acquired Respiratory Syndrome	−222.05	42	Baylisascariasis	−155.26	42	−1
Old/New World Screwworm	−245.17	43	Toxocariasis	−157.14	43	−5
Western Equine Encephalitis	−250.06	44	Cutaneous larva migrans	−158.61	44	−5
Anaplasmosis/Canine granulocytic anaplasmosis	−256.71	45	Cysticercosis/Taeniasis	−168.25	45	12*
Typhus	−272.70	46	Western Equine Encephalitis	−185.49	46	−2
Japanese encephalitis	−273.33	47	Severe Acquired Respiratory Syndrome	−194.66	47	−5
Monkeypox	−279.78	48	Hepatitis A	−205.98	48	6
Trichinosis	−316.26	49	Japanese encephalitis	−230.44	49	−2
Babesiosis	−316.48	50	Lassa fever	−231.04	50	−10*
Venezuelan Equine Encephalitis	−329.70	51	Old/New World Screwworm	−251.11	51	−8
Yellow Fever	−330.35	52	Monkeypox	−274.35	52	−4
Cholera	−342.29	53	Venezuelan Equine Encephalitis	−279.00	53	−2
Hepatitis A	−359.51	54	Yellow Fever	−303.53	54	−2
Bovine Tuberculosis	−370.50	55	Trichinosis	−338.97	55	−6
Rift Valley fever	−372.81	56	St. Louis encephalitis	−363.37	56	6
Cysticercosis/Taeniasis	−443.42	57	Cyclosporiasis	−363.46	57	2
Coccidioidomycosis	−459.90	58	La Crosse encephalitis	−394.32	58	3
Cyclosporiasis	−490.94	59	Bovine Tuberculosis	−397.95	59	−4
Dengue fever	−520.64	60	Cholera	−416.70	60	−7
La Crosse encephalitis	−589.41	61	Dengue fever	−422.66	61	−1
St. Louis encephalitis	−597.52	62	Rift Valley fever	−425.87	62	−6

* Diseases that deviated by more than 10 ranked positions between countries.

### Disease Priority Lists

The final ranking of diseases by their overall CA-derived scores is presented in Table 8. The higher the score, the higher the ranking on the priority list. The range in the overall scores by diseases differed between Canada and the US and correlates with the part-worth utility values derived by country (Tables 1, 2, 3). As part-worth utility values are interval data, the overall scores cannot be directly compared both within and between countries [24]. We can, however, compare the difference in disease ranking as an overall measure of proximity of diseases both within and between countries. Although differences in disease ranking were observed between countries, the majority of diseases (77%) were within ten ranked positions of each other indicating a general consensus in ranks between countries. Diseases of high priority generally exhibited high incidence, high case-fatality, severe symptoms, prolonged duration of illness, high transmission potential and are increasing or emerging in humans and animals, though it was not necessary to exhibit all of these characteristics to be prioritized (e.g., *Nipah virus encephalitis*, *Ebola* and *Marburg* do not occur naturally in North America while *H1N1 Influenza* has a low case-fatality rate). Diseases of low priority generally comprised rare diseases or diseases with a large proportion of asymptomatic cases, low case-fatality, short duration of illness, mild symptoms, stable disease trend and low transmission potential in humans and animals.

Canadians considered *Giardiasis*, *Salmonellosis*, *Lassa fever*, *Cryptosporidiosis*, *Toxoplasmosis* and *Botulism* of higher priority than Americans. Conversely, Americans considered *Babesiosis, Anaplasmosis, Paralytic shellfish poisoning, Coccidioidomycosis, Cysticercosis, Hantavirus pulmonary syndrome, Rocky Mountain spotted fever, Typhus,* and the *plague* of higher priority than Canadians. This was due to regional differences in disease incidence (*Anaplasmosis*, *Rocky Mountain spotted fever* and *Babesiosis* do not occur naturally in Canada but are endemic in the US), differences in disease trend (*Paralytic shellfish poisoning* has been increasing in the US but is stable in Canada) or differences in the part-worth utility values within disease criteria by country (Tables 1, 2, 3).

## Discussion

Disease prioritization has engaged the interest of numerous research groups recently [4], [6], [12], [13], [15]–[18]. As zoonotic outbreaks are a significant public health burden in North America [25]–[27], and with decreasing resources available for their prevention and control, a strategic framework for the prioritization of zoonoses would be valuable. We present on the novel use of a quantitative methodology, CA, for the prioritization of zoonoses in North America. There are a number of unique features in our study that overcome constraints in traditional prioritization methodologies. Our method fits a statistical model to a robust experimental design to generate relative weighted scores for disease criteria and their levels thus overcoming the limitation of a subjective weighting process for disease criteria by expert panels [4], [6], [13], [16], [18] or an arbitrary scoring system for levels [4], [6], [8]–[13], [16], [18]. Because CA generates weighted scores by assessing disease combinations rather than disease criterion separately, our scores are relative weighted scores that do not assume disease criteria are independent or of equal importance [4], [6], [8]–[13], [16], [18]. The study explored preference for disease criteria, rather than actual diseases; thus, individuals were forced to prioritize on the basis of scientific information, eliminating biases associated with disease names. We elicited preferences by choice rather than by ranking or rating diseases allowing for a more intuitive approach to expressing preferences, particularly when choices are similar [17].These features of the CA trade-off methodology brings additional value to the disease prioritization process compared to traditional methods.

Perhaps the most distinct feature of this study was the ability to engage wide social participation from the public. This has not been previously conducted and understanding the perception of the public may offer healthcare professionals the opportunity to improve public education and risk communication. Our CA models indicate individuals with no prior knowledge or experience in prioritizing zoonoses were capable of producing meaningful results with a satisfactory model fit. Part-worth utility values exhibited face validity with a stronger preference for salient disease criteria levels than non-salient alternatives. The degree to which each disease criterion contributed to the overall decision to prioritize also demonstrated face validity and are consistent with findings from similar studies [4], [6], [13], [16]–[18]. The priority lists derived from applying part-worth utility values from the CA models to actual diseases produced a list of diseases that were reasonable, from a public perspective, for prioritization. More similarities than differences were observed in the strength of preference in disease criteria importance scores between countries suggesting general agreement in disease prioritization between Canadians and Americans.

There are some limitations associated with this study. The response rate was low given the length and technical content of the survey, we also used a pre-screened panel to recruit participants; the study populations were therefore highly educated compared to the national populations and the results may not be truly representative of the general public. However, public education and risk communication has the highest impact on those who are educated and actively involved in the community, thus, our study populations likely represent the target population for public health intervention. There was a marked difference in the response rate between the Canadians and Americans, although the cause for this difference is unknown as recruitment strategies and survey design were identical between countries. It is important to note that while response rates differed dramatically, the study populations did not deviate considerably from their respective national populations or from each other in terms of gender, age and education. The difference in response rate is therefore unlikely to have resulted in substantial differences in the results between countries.

Our percent certainty model fit was 79.4% for both countries; an opportunity therefore exists to improve the models further to estimate more precise part-worth utility values. Marginal changes in part-worth utility values may have a substantial impact on the relative ranking of diseases on the priority list; thus, our current priority lists are only acceptable if we accept the current model fit. Nonetheless, this is the first disease prioritization study to validate disease criteria scores against respondent choices and may serve as a standard for prioritization methodologies. We also acknowledge that not all of the 59 disease criteria identified from focus groups was considered in this study [7], the inclusion of additional criteria would have an impact on the relative ranking of diseases. The current priority lists are therefore only applicable to the 21 disease criteria in the study, in spite of this; we believe these criteria capture some of the most relatively important aspects for priority setting.

Finally, there are multiple objectives for prioritizing zoonoses (e.g. prioritizing for research, regulation, control, prevention, management, vaccination, diagnosis, cost-effective and surveillance), although we specified to participants to prioritize “for policy implementation for the control and prevention of zoonoses”, they may have prioritized with another objective in mind. There is no way to measure this type of error and we assume that participants were consistent in their objective to prioritize. Further, we asked participants “which of the following diseases should be prioritized” with each choice task set, which may be ambiguous in the face of multiple competing diseases. Although we did state in the instructions (not presented) to “select one of the five disease profiles that characterizes a disease that, in your opinion, should be prioritized above the others”, we could have reinforced this by explicitly specifying, “which of the following diseases should receive the highest priority” with each choice task set. We assume that participants understood the research question and were consistent in their interpretation.

In conclusion, we describe the first zoonotic disease prioritization exercise, involving public participation, in North America. Given the established quantitative approach, robust experimental design, satisfactory model fit and reasonable disease criteria level scores [24], [34], we have demonstrated CA as a potential tool for the prioritization of zoonoses. Periodical updating of criteria levels to match current disease trends will allow for the revision of disease priority lists that will reflect the most current state. Finally, the application of this methodology is versatile and not limited to zoonoses or North America; thus, other prioritization exercises may consider this approach with different criteria inputs.
